# Impact of Elexacaftor–Tezacaftor–Ivacaftor Therapy on Body Composition, Dietary Intake, Biomarkers, and Quality of Life in People with Cystic Fibrosis: A Prospective Observational Study

**DOI:** 10.3390/nu16193293

**Published:** 2024-09-28

**Authors:** Francisco Hevilla, Nuria Porras, María Victoria Girón, María García-Olivares, Marina Padial, Francisco José Sánchez-Torralvo, Casilda Olveira, Gabriel Olveira

**Affiliations:** 1 Departamento de Medicina y Dermatología, Facultad de Medicina, University of Malaga, 29010 Malaga, Spain; 2 Instituto de Investigación Biomédica de Málaga (IBIMA), Plataforma Bionand, 29010 Malaga, Spain; 3 Unidad de Gestión Clínica de Endocrinología y Nutrición, Hospital Regional Universitario de Málaga, 29007 Malaga, Spain; 4 Unidad de Gestión Clínica de Neumología, Hospital Regional Universitario de Málaga, 29010 Malaga, Spain; 5 Centro de Investigación Biomédica en Red de Diabetes y Enfermedades Metabólicas Asociadas (CIBERDEM), Instituto de Salud Carlos III, 28029 Madrid, Spain

**Keywords:** cystic fibrosis, elexacaftor–tezacaftor–ivacaftor (ETI), body composition, bioelectrical impedance analysis (BIA), skinfold thickness measurement, dietary surveys

## Abstract

**Background:** The combination of elexacaftor–tezacaftor–ivacaftor modulators (ETI) has improved clinical outcomes for people with cystic fibrosis (pwCF). **Objectives:** This study aimed to evaluate changes in nutritional and morphofunctional assessments, as well as anxiety, depression symptoms, and quality of life, in pwCF after starting ETI therapy. **Methods:** This was a prospective observational study. We measured body composition (fat mass [FM] and fat-free mass [FFM]) using bioelectrical impedance analysis (BIA) and skinfold thickness measurements (SMs). We also assessed hand grip strength, dietary intake via surveys, blood and stool biomarkers, symptoms of anxiety and depression using the Hospital Anxiety and Depression Scale [HADS], and quality of life through the Cystic Fibrosis Questionnaire—Revised (CFQR). **Results:** A total of 31 pwCF were evaluated. Significant improvements were observed in respiratory function and quality of life, alongside an average weight increase of approximately 5 kg (60% FM and 40% FFM). The prevalence of malnutrition, based on BMI and the FFM index, decreased significantly, while the rate of overweight/obesity increased. Biomarker analysis indicated better nutrient absorption and reduced intestinal inflammation, as evidenced by significant changes in faecal calprotectin, nitrogen, and fat levels, as well as blood lipid and vitamin profiles. **Conclusions:** Despite a reduction in caloric intake, an increase in weight was observed one year after initiating ETI. This increase was attributed to gains in both FM and FFM, suggesting improved metabolic efficiency and nutrient absorption. Both SM and BIA were found to be useful assessment tools. These findings indicate the need to modify the nutritional approach, focusing on the quality rather than the quantity of intake, and aiming for an appropriate body composition (FFM) rather than solely focusing on BMI.

## 1. Introduction

Cystic fibrosis (CF) is a disease caused by the alteration of a single gene, the CFTR gene (cystic fibrosis transmembrane conductance regulator). The protein encoded by the CFTR gene functions as a chloride channel, and mutations result in a defect in chloride transport in the epithelial cells of the respiratory, hepatobiliary, gastrointestinal, reproductive, pancreatic, and sweat gland systems. Due to the multitude of organs and systems it affects, CF is a complex and multisystemic disease that requires a multidisciplinary approach [1].

Currently, the triple combination of CFTR modulators, elexacaftor–tezacaftor–ivacaftor (ETI), has become the new standard of care for people with CF (pwCF) carrying at least one F508del CFTR variant [2,3]. Data from randomised clinical trials revealed improvements in respiratory outcomes (respiratory symptoms, lung function, exacerbations) and body mass index [2,4,5], which were largely confirmed in real-world studies [6,7,8].

CF-associated poor nutritional status is a multifactorial syndrome caused by nutrient malabsorption, inadequate nutrient intake, decreased appetite, and higher energy needs [9]. Poor nutritional status is linked to worse pulmonary function and increased mortality in pwCF. Improved nutritional CF care significantly reduced the rate of malnutrition before the generalisation of ETI modulator treatment; however, figures close to 25% continued to be reported in both children and adults [10,11].

The guidelines emphasise the need to conduct a longitudinal assessment of body composition to obtain estimates of fat mass (FM) and fat-free mass (FFM) [3,12] because their association with respiratory outcomes is stronger than for BMI alone, and because a normal or high BMI can mask low FFM [13,14,15]. This could be important in the context of CFTR modulator therapy [3,15]. The choice of body composition (BC) method should be guided based on availability, resources, technical factors, and clinical factors [12].

Weight improvements have been published in the vast majority of real-life studies after ETI treatment [6,7,8]. However, few have evaluated the change in BC [11,16,17,18,19], and the findings regarding which components—fat mass and/or fat-free mass—increase remain inconsistent. Additionally, no study has examined the use of anthropometric methods, such as skinfold thickness measurements (SMs), in patient follow-up. These could be particularly useful in settings where more advanced techniques like bioelectrical impedance analysis (BIA), dual-energy X-ray absorptiometry (DXA), ultrasonography, or computed tomography (CT) are unavailable. The mechanisms behind weight gain after ETI remain unclear and are likely multifactorial. Improvements in intestinal absorption [20] and changes in biomarkers such as lipids and vitamins have been observed [8,20]. Moreover, only one study has prospectively evaluated dietary intake [21]. It is important to note that dietary intake may be influenced by anxiety and depression symptoms, which are common in pwCF and may, in turn, affect their quality of life in all its dimensions [22]. To date, no study has holistically evaluated all these aspects.

With this background, the objective of this study was to prospectively and comprehensively assess mid-term changes in morphofunctional evaluation, including body composition (via BIA and SM), hand dynamometry, prospective dietary surveys, blood and stool biomarkers, as well as symptoms of anxiety and depression, and quality of life in adults with cystic fibrosis (pwCF) following the initiation of ETI therapy.

## 2. Materials and Methods

Design: Prospective observational study of routine clinical practice. Adult pwCF from the Cystic Fibrosis Adult Unit who began taking elexacaftor–tezacaftor–ivacaftor were eligible for inclusion and were studied at baseline and one year after starting ETI treatment.

### 2.1. Morphofunctional Assessment

#### 2.1.1. Body Composition

Weight and BC (phase angle, fat-free mass, and fat mass) were assessed using a BIA scale (TANITA MC980MA, TANITA Corporation, Tokyo, Japan), and height was obtained using a stadiometer (Holtain Limited, Crymych, UK). The skinfolds measured were the triceps, biceps, subscapular, and supra-iliac, using a Holtain constant pressure caliper (Holtain Limited, Crymych, UK). The same investigator (N.P.) performed the measurements in triplicate for each of the skinfolds assessed, and the mean was calculated. FM and FFM were estimated according to the formulas of Siri and Durnin [23,24]. The FFM index (FFMI) was calculated using anthropometry and BIA. The prevalence of malnutrition was determined according to the criteria: <15 kg/m^2^ for women or <17 kg/m^2^ for men.

#### 2.1.2. Muscle Strength

Muscle strength was assessed using a Jamar dynamometer (Asimow Engineering Co., Los Angeles, CA, USA) on the dominant hand. The measurement was repeated three times, and the mean was calculated.

#### 2.1.3. Dietary Questionnaire

A 4-day prospective dietary questionnaire was completed. The data provided were analysed using a computer application designed by our group for this purpose (Dietstat^®^, FIMABIS, Málaga, Spain) [25] and the food composition tables of Jiménez and Mataix [26,27] and BEDCA [28] were used. In cases where patients were receiving medical nutritional therapy through oral nutritional supplements or enteral nutrition via a feeding tube, the composition was also included in the database of the DIETSTAT programme and accounted for.

#### 2.1.4. Laboratory Measurements

A complete blood test was performed to assess haemogram, coagulation, and biochemical values, including albumin, prealbumin, *C*-reactive protein, immunoglobulins (with an autoanalyzer), glycated haemoglobin (following the international recommendations for standardisation of the HbA1c measurement [29]), and fat-soluble vitamins (A, D, and E). Vitamins A and E were measured using High-Performance Liquid Chromatography (HPLC, Agilent 1200, Bio-Rad, Hercules, CA, USA) and vitamin D was assessed through an electrochemiluminescent immunoassay (Modular E-170, Roche Diagnostics, Mannheim, Germany). A 72 h stool sample was collected for the quantitative measurement of faecal fat and nitrogen by means of a spectrophotometer technique (near-infrared reflectance analysis), as well as elastase 1 (ELISA ScheBo, Biotech AG, Gießen, Germany)) and calprotectin (ELISA Calprest^®^ Eurospital, Trieste, Italy).

### 2.2. Quality of Life—CFQR14+ (Spain)

It consists of 50 items divided into twelve domains. Scores range from 0 to 100, with higher scores indicating better HRQoL [30].

### 2.3. Assessment of Respiratory Status

The exacerbations recorded during the annual examination were assessed, considering those occurring in the year prior to the evaluation. They were classified into mild/moderate or severe (suggestive symptoms that worsen and require hospitalisation and/or intravenous antibiotics on an outpatient basis). Chronic colonisation was defined as the presence of three or more consecutive positive cultures for the same pathogen over a period of at least twelve months, with samples taken at least one month apart. Moreover, patients underwent forced spirometry following the guidelines of the Spanish Society of Pulmonology and Thoracic Surgery (SEPAR), determining the values of forced vital capacity (FVC), forced expiratory volume in 1 s (FEV1), and the ratio between both (FEV1/FVC) [31]. The values were expressed in absolute terms in ml and as percentages according to a reference population. We also evaluated the E-FACED-Score [32].

### 2.4. Hospital Anxiety and Depression Scale

It is a 14-item instrument: 7 questions measure depression and 7 measure anxiety. Respondents indicated the severity of each symptom over the past week. The maximum score is 21. A score of less than 8 is considered a negative result in the screening [33].

### 2.5. Statistical Analysis

Quantitative variables were expressed as the mean ± standard deviation, and the distribution was assessed using the Shapiro–Wilk test. Differences between quantitative variables were analysed using the paired Student’s *t*-test or Wilcoxon test. Comparisons between the three groups were performed using ANOVA with Bonferroni post hoc tests or Kruskal–Wallis tests. The associations of the variables were evaluated by estimating the Pearson or Spearman correlation coefficient according to normality. For the comparison of proportions of qualitative variables, such as the percentage of malnourished and obese individuals before and after ETI, McNemar’s test was used. For calculations, significance was set at *p* < 0.05 for two-tailed tests. Data analysis was performed with the JAMOVI program (version 2.3.28).

### 2.6. Ethics

All subjects gave their informed consent to participate in the study. The study was conducted in accordance with the Declaration of Helsinki, and the protocol was approved by the Research Ethics Committee of Málaga on 30 March 2021.

## 3. Results

A total of 34 adults with CF who initiated ETI were recruited, among which three were excluded during follow-up (one for lack of compliance and two for voluntarily undertaking a low-calorie diet to lose weight during the follow-up period). The patients began treatment with ETI at a mean age of 30.7 ± 9 years, and the average follow-up time was 1 year and 1 month. F508del homozygotes made up 29% of the study cohort, F508del heterozygotes 58%, and four patients (13%) received treatment as compassionate use (without the F508del mutation). Twenty-seven (87%) had pancreatic insufficiency, eight (26%) had carbohydrate intolerance, and seven had CF-related diabetes (22.5%), among which six received insulin treatment (19%). All patients treated with insulin continued the treatment after one year, and one person with carbohydrate intolerance achieved a normal curve without changes in the rest. Seven subjects were receiving oral nutritional supplements (ONSs) before treatment, and one person was on enteral nutrition via gastrostomy. After one year, only two continued ONSs, and gastrostomy was removed 6 months after starting ETI. Seven patients had HADSa (Hospital Anxiety and Depression Scale anxiety subscale) scores above 8, and after one year, only five remained the same; two had HADSd (depression subscale) scores above 8, and these remained unchanged after one year.

Table 1, Table 2, Table 3, Table 4 and Table 5 summarise the changes before and after treatment with ETI. We observed significant differences in respiratory spirometric parameters, exacerbations, and chronic colonisation by Staphylococcus aureus (Table 1); weight and body composition (BMI, FM, FFM, FFM index, and the percentage of people with malnutrition, overweight/obesity, and FFM malnutrition), both measured by SM and BIA (Table 2); calories from dietary intake and grams of macronutrients (total and monounsaturated fats and carbohydrates) (Table 3); LDL cholesterol, HbA1C, immunoglobulin G, vitamin A, nitrogen, fat, and faecal calprotectin (Table 4); and quality of life dimensions: vitality, body, eating, treatment, health, weight, and respiratory (Table 5).

The weight gain was significantly greater (*p* < 0.05) in patients with CF with a BMI less than 18.5 kg/m² at the beginning (*n* = 7: 7.2 ± 5 kg) and also in those with overweight or obesity (OW/O) (*n* = 5: 9.7 ± 5.4 kg) compared to those with normal weight (*n* = 19: 2.7 ± 2.7 kg).

We observed significant negative correlations between the increase in weight (*p* = 0.043), FM (*p* = 0.023), and FFM (*p* = 0.033) measured by skinfolds and the baseline FEV1/FVC ratio, as well as positive correlations between the number of severe exacerbations in the year prior to starting ETI and the increase in FFM (BIA) (*p* = 0.022) and almost significant FFM by skinfolds (*p* = 0.08).

## 4. Discussion

In our study, we observed an average weight increase of approximately 5 kg during the first year of follow-up, consisting of 60% fat mass and 40% fat-free mass. Both skinfold measurements and bioelectrical impedance analysis were useful, yielding very similar results. These changes were accompanied by improvements in pulmonary function, quality of life, a decrease in caloric intake, and enhancements in biomarkers related to absorption and intestinal inflammation.

### 4.1. Morphofunctional Assessment

The prevalence of malnutrition (according to BMI) in our series decreased from 22.6% to 3.2%, while the prevalence of overweight/obesity (OW/O) increased from 16% to 26%. Both bioelectrical impedance (BIA) and skinfold measurements (SMs) are non-invasive, safe, quick, and accessible methods at the point of care, providing immediate results [14,34]. Using these techniques, the percentage of pwCF with FFM malnutrition decreased significantly, from 35% to 16% (estimated by skinfolds) and from 29% to 10% (estimated by BIA). The slight differences may be due to BIA generally overestimating the FFM compared to skinfold measurements in individuals with CF and bronchiectasis [14,34]. Increased adiposity along with low FFM may be even more detrimental to pulmonary function in pwCF [35].

In a large series of 434 patients with CF after ETI initiation, the percentage of patients with malnutrition (body mass index <18.5 kg/m²) decreased from 38.6% to 11.3% at 12 months (*p* = 0.0001). The weight increased during the first year and then stabilised [7]. In the Petersen series, decreases were observed in the rates of underweight (7.5% to 2.2%) with increases in rates of overweight (19.4% to 31.3%) and obesity (7.5% to 9.7%) [8]. Similarly, in our group, Proud et al. demonstrated an increase in both FFM and FM using BIA, with mean increases of 2.5 kg and 2.1 kg, respectively, seven months after initiating ETI [19].

However, not all studies have observed increases in FFM. Grancini et al., in a group of 24 patients with CF-related diabetes, demonstrated that, after six months of treatment, FM (measured by BIA) significantly increased by a median value of 1.6 kg, but without a significant increase in FFM [16].

Knott-Torcal et al., in 36 pwCF, also observed a significant increase in BMI after six months of treatment, as well as an increase in FM and visceral fat area, with a trend towards an increase in FFM (by approximately 600 g), though this did not reach statistical significance [17]. This same group published, in 26 adult subjects, changes in body composition using CT scans at the level of the 12th dorsal vertebra and observed an increase in total body area, driven by increases in total FM, subcutaneous fat, visceral fat, and intermuscular fat (all with significant differences). The only muscle compartment that showed an increase after treatment was very-low-density muscle, suggesting an increase in myosteatosis [18].

In a retrospective study, an automated analysis of body composition on the chest CT scans of 66 adult patients with CF was performed. They observed marked increases in all adipose tissue ratios, including the total adipose tissue ratio (+46.21%); conversely, only small (but statistically significant) increases in the muscle ratio (+1.63%). Study participants who were initially categorised as underweight experienced more pronounced effects on the total adipose tissue ratio, while gains in muscle ratio were equally distributed across BMI categories [11]. In our series, in contrast, both patients with CF with low BMI at initiation and those with overweight/obesity (OW/OB) gained more weight compared to those with normal BMI (who showed the least total weight gain).

Concomitant with the changes in body composition, we observed significant improvements in spirometric parameters, a reduction in both total and severe respiratory exacerbations, and a decrease in chronic bacterial colonisation. Having a higher number of severe exacerbations prior to ETI treatment was associated in our sample with an increase in FFM in the first year, as well as a lower FEV1/FVC ratio with greater weight gain in FM and FFM. Stewart et al. also observed in a series of young adults that younger age and more frequent prior pulmonary exacerbations had the strongest relationships to 6-month increases in BMI [36]. In the study by Navas-Moreno, higher levels of very low-density muscle prior to treatment were associated with lower final FEV1 and less improvement in FEV1 after therapy [18].

While all studies document weight and fat mass increases, discrepancies in FFM changes among studies may be due to various factors: differences in pwCF populations (with a varying severity of disease at baseline), measurement techniques of BC, and levels of physical exercise or training. In a series by Gruber et al., it was observed that, after ETI treatment, pwCF had a significant increase in steps/day (+25%) [37]. Increased physical activity could lead to improvements in FFM. In a Danish prospective study of 229 pwCF evaluating the impact of CFTR modulators on exercise capacity using the cardiopulmonary exercise test (CPET), a significant increase in oxygen uptake was observed; however, the change was not clinically relevant and considerable variability was observed in the sample. Changes in FEV1% and BMI were able to explain some of the differences [38]. In another series with long-term prospective follow-up evaluating exercise capacity in pwCF (measured by incremental cycle test), an improvement was only observed in pwCF using ETI, whereas pwCF not using ETI showed a small decrease; however, overall, the impact of ETI on all aspects of physical fitness was small [39]. It is likely that CFTR modulators alone are not sufficient for recovering physical deconditioning, but should be supplemented with physical activity and respiratory physiotherapy [40]. Unfortunately, we did not assess the changes in the physical activity levels of our patients, although indirectly, we observed a significant increase in the vitality dimension of the CFQR test. Conversely, we did not observe changes in strength as measured by hand grip dynamometry.

### 4.2. Serum and Faecal Biomarkers

The causes underlying weight increases following modulator therapies are not well understood but could be multiple, including a reduction in energy expenditure and systemic inflammation, improvement in glycaemic control, changes in dietary intake, and changes in fat absorption, gut inflammation, and microbiota modifications [7,8,16,20].

In our study, we observed significant decreases in faecal calprotectin levels, as well as nitrogen and fat in the stools, while LDL cholesterol and vitamin A levels increased significantly, with vitamin E, albumin, and total cholesterol levels nearing significance. Additionally, the dimensions of quality of life, body image, eating problems, and weight improved, indicating better nutrient absorption and reduced intestinal inflammation. Statna et al. also observed lower pancreatic enzyme replacement requirements and improved defecation in adult patients with CF on ETI, along with increased albumin and prealbumin levels [20]. In Burgel et al.’s series, significant increases in serum concentrations of vitamins A and E were noted [7], and Petersen et al. and Docherty et al. reported increases in total cholesterol and LDL [8,41].

Numerous studies have demonstrated improvements in metabolic control following ETI, with a reduction or even discontinuation of insulin therapy [7,8,16,42]. In our series, metabolic control also improved with significant decreases in HbA1c by 0.2%, although insulin was not discontinued in any patient. The underlying mechanism is still not completely understood and could be related to a decreased inflammatory state, improved insulin sensitivity, and better beta-cell function.

### 4.3. Dietary Intake

These changes occurred in our series despite observing a decrease in total caloric dietary intake and grams of macronutrients (except fibre), but not in their percentage. Of the seven patients taking oral nutritional supplements prior to ETI initiation, only two maintained their intake after one year, and the only person with a gastrostomy discontinued it within the first six months. These findings align with the series by Caley et al., in a group of 40 adult pwCF, where weight increased despite a reduction in caloric intake [21]. In Burgel et al.’s series, a 50% decrease in the number of patients using oral nutritional supplements and the discontinuation of the enteral tube feeding in most patients was observed over the first year following ETI initiation [7].

### 4.4. Psychological Symptoms

Depressive and/or anxious symptoms can also influence intake as they can worsen the perception of quality of life and adherence to treatment [22,43]. Although CFTR modulator therapies provide hope for improving clinical outcomes, worsening depression and anxiety occur in some patients when starting these novel agents [44]. We did not observe changes in depression and anxiety symptoms or the corresponding dimension of the CFQR. In a study of 100 pwCF, no changes in symptoms were observed; however, a quarter of patients did display a change in psychiatric medications [43].

### 4.5. Limitations

Although some results did not reach statistical significance, the observed changes in body composition, respiratory function, quality of life, blood and stool biomarkers, and dietary caloric intake provide meaningful clinical insights for healthcare professionals managing CF patients post-ETI therapy. These findings underscore the importance of adopting a holistic approach to care.

The strengths of this study include its prospective nature and the use of various techniques for a comprehensive assessment of morphofunctional changes (body composition by BIA and SM, hand grip dynamometry, prospective dietary survey, blood and stool biomarkers, quality of life) as well as anxiety and depression symptoms in adult pwCF. This study is limited by its single-centre observational design and relatively small sample size, which increases the risk of Type 1 and Type 2 errors and limits the generalisability of the results. Functionality was also assessed using only handgrip dynamometry.

## 5. Conclusions

Adults with CF one year after initiating ETI treatment increased weight at the expense of FM and FFM. Both SM and BIA were useful for the longitudinal monitoring of body composition. These changes were parallel to improvements in pulmonary function, quality of life, biomarkers related to absorption and intestinal inflammation, and a decrease in caloric intake and the need for oral nutritional supplements. These results emphasise the importance of monitoring patients with CF with a focus on the quality rather than quantity of intake, and on body composition and exercise rather than BMI. Future studies with larger cohorts are essential to validate these results and further elucidate the mechanisms underlying these changes.

## Figures and Tables

**Table 1:** General characteristics and respiratory status.

	Pre ETI (*n* = 31)	Post ETI (*n* = 31)	*p*-Value
Age	30.7 (±9)	31.9 (±9)	<0.001
Pancreatic insufficiency (n, %)	27 (87%)	27 (87%)	1
Endocrine pancreas			
Glucose intolerance	8 (26%)	7 (22.5%)	0.9
CF related diabetes	7 (22.5%)	7 (22.5%)	1
Insulin therapy	6 (19%)	6 (19%)	1
Mild exacerbations	1.1 (±1.2)	0.4 (±0.7)	0.004
Severe exacerbations	0.6 (±0.9)	0.1(±0.3)	0.021
FEV_1_ (mL)	1849.0 (±977.3)	2105.1 (±1012.8)	0.001
% FEV_1_	51.4 (±22.2)	58.5 (±23.3)	<0.001
FVC (mL)	2711.0 (±1095.6)	3043.6 (±1126.7)	<0.001
% FVC	61.3 (±19.0)	68.0 (±19.0)	<0.001
FEV_1_/FVC	0.66 (±0.12)	0.67 (±0.11)	0.289
Chronic colonisation of the respiratory tract (n, %)			
Haemophilus influenzae	2 (6%)	2 (6%)	1
Pseudomonas aeruginosa	12 (35%)	8 (23.5%)	0.125
Staphylococcus aureus	18 (53%)	12 (35%)	0.031
E-FACED Score	3.6 (±20.1)	3.1 (±15.3)	0.100

Data are shown as mean (±standard deviation). Elexacaftor–tezacaftor–ivacaftor (ETI); FEV_1_: forced expiratory volume in 1 s; FVC: forced vital capacity. E-FACED is a scoring system used to predict the risk of exacerbations in patients with bronchiectasis: (exacerbations, forced expiratory volume in 1 s (FEV1), age, colonisation with pseudomonas aeruginosa, radiological extent of the disease, and dyspnoea).

**Table 2:** Body composition and muscle strength.

	Pre ETI (*n* = 31)	Post ETI (*n* = 31)	*p*-Value
Weight (kg)	59.2 (±15.2)	64.1 (±17.1)	<0.001
BMI (kg/m^2^)	21.5 (±3.8)	23.2 (±4.2)	<0.001
% low BMI	22.6	3.2	0.020
% overweight/obesity	16.1	25.8	0.020
% FM_SM_	20.7 (±8.7)	23.3 (±9.5)	<0.001
FM _SM_ (kg)	12.3 (± 6.5)	14.9 (±7.6)	<0.001
% FM _SM_	79.3 (±8.7)	76.7 (±9.5)	<0.001
FFM _SM_ (kg)	46.0 (±11.1)	48.0 (±12.0)	<0.001
FFMI _SM_ (kg/m^2^)	16.8 (±2.6)	17.4 (±2.7)	<0.001
% low FFMI_SM_	35.5	16.7	0.030
% FM_BIA_	22.9 (±11.4)	24.5 (±7.8)	0.326
FM_BIA_ (kg)	13.5 (±6.8)	16.8 (±8.3)	<0.001
%FFM_BIA_	77.2 (±11.5)	75.3 (±8.0)	0.210
FFM (kg) _BIA_	46.5 (±10.8)	48.6 (±11.5)	<0.001
FFMI _BIA_	16.9 (±2.3)	17.6 (±2.4)	<0.001
% low FFMI_BIA_	29	10.3	0.063
Phase angle (°)	5.3 (±0.8)	5.5 (±0.8)	0.099
Triceps ST (mm)	12.6 (±7.0)	15.1 (±7.8)	<0.001
Biceps ST (mm)	7.2 (±4.5)	8.9 (±5.5)	0.028
Subscapular ST (mm)	11.5 (±5.9)	13.7 (±6.8)	0.002
Abdominal ST (mm)	15.7 (±9.1)	17.7 (±9.3)	0.067
Suprailiac ST (mm)	10.6 (±7.5)	12.6 (±7.4)	0.029
MUAC (cm)	25.9 (±3.6)	27.2 (±4.1)	0.002
Max. dynamometry (kg)	34.1 (±12.7)	34.2 (±11.9)	0.884
Mean dynamometry (kg)	33.0 (±12.5)	33.3 (±11.7)	0.645

Data are shown as the mean (±standard deviation). Elexacaftor–tezacaftor–ivacaftor (ETI); BMI: body mass index; low BMI: BMI < 18.5 kg/m^2^; overweight: BMI ≥ 25 and <30; obesity: BMI ≥ 30; FM: fat mass; _SM_: estimated according to skinfold thickness measurements; FFM: fat-free mass; FFMI: fat-free mass index; low FFMI: <17 kg/m^2^ in men and <15 kg/m^2^ in women; _BIA_: measured by bioelectrical impedance (BIA); ST: skinfold thickness; MUAC: mid-upper arm circumference; Max. dynamometry: maximum hand grip dynamometry.

**Table 3:** Dietary survey.

	Pre ETI (*n* = 31)	Post ETI (*n* = 31)	*p*-Value
Energy (kcal)	2621.0 (±456.3)	2355.9 (±369.7)	0.022
Protein (g)	109.6 (±29.4)	100.0 (±24.0)	0.064
Protein (% energy)	16.7 (±2.6)	16.9 (±2.6)	0.218
Total fat (g)	126.3 (±30.6)	107.6 (±22.7)	0.006
Total fat (% energy)	42 (±5.8)	39.0 (±4.2)	0.216
Saturated fat (g)	29.8 (±6.4)	26.9 (±6.1)	0.073
Saturated fat (% of fat)	28.0 (±5.4)	29.1 (±6.9)	0.616
Monounsaturated fat (g)	55.4 (±14.6)	47.2 (±11.2)	0.012
Monounsaturated fat (% of fat)	52.8 (±4.4)	51.7 (±6.1)	0.615
PUFAs (g)	19.9 (±10.4)	16.1 (±7.0)	0.112
PUFAs (% of fat )	18.6 (±7.2)	18.1 (±5.7)	0.834
PUFA-Omega 3 (g)	2.3 (±1.7)	2.3 (±1.3)	0.906
Carbohydrate (g)	261.1 (±48.8)	245.1 (±49.1)	0.035
Carbohydrate (% energy)	39.8 (±5.9)	41.1 (±5.1)	0.439
Fiber (g)	19.7 (±8.0)	19.0 (±8.7)	0.575
Medical nutritional therapy (n, %)			0.031
Tube feeding	1 (2.9%)	0 (0%)	
Oral nutritional supplements	7 (20%)	2 (5.8%)	

Data are shown as mean (±standard deviation. This refers to the average daily intake of energy and macronutrients. Elexacaftor–tezacaftor–ivacaftor (ETI); PUFAs: polyunsaturated fatty acids.

**Table 4:** Blood and stool test parameters.

	Pre ETI (*n* = 31)	Post ETI (*n* = 31)	*p*-Value
Neutrophils (×10^3^/µL)	5.2 (±2.7)	4.4 (±3.0)	0.350
% Neutrophils	60.3 (±10.7)	56.2 (±11.0)	0.15
% Prothrombin time	93.1 (±14.3)	98.5 (±12.3)	0.172
Total cholesterol (mg/dL)	146.1 (± 34.9)	158.3 (±44.8)	0.098
HDL (mg/dL)	54.1 (±17.3)	52.8 (± 13.6)	0.639
LDL (mg/dL)	84.7 (±26.4)	101.7 (±34.5)	0.023
Triglycerides (mg/dL)	77.2 (±22.3)	89.9 (±33.1)	0.192
HbA1c (%)	5.9 (±0.8)	5.6 (±0.5)	0.017
Albumin (g/dL)	3.8 (±0.5)	3.9 (±0.3)	0.441
Prealbumin (mg/dL)	23.4 (±4.9)	26.4 (±7.7)	0.048
*C*-reactive protein (mg/dL)	11.7 (±16.7)	4.2 (±4.2)	0.130
Immunoglobulin G (mg/dL)	1606.8 (±393.5)	1392.5 (±324.1)	0.002
Vitamin A (µg/dL)	48. 1 (± 15.4)	55.7 (± 16.3)	0.048
Vitamin D3 (ng/mL)	36.1 (±16.2)	33.5 (±12.9)	0.570
Vitamin E (µg/dL)	1185.9 (± 354.0)	1277.6 (± 419.1)	0.389
Zinc (µg/dL)	77.3 (±18.1)	84.7 (±9.1)	0.153
Faecal nitrogen (g)	7.2 (±5.0)	3.7 (±2.6)	0.021
Faecal fat (g)	11.7 (±7.0)	8.3 (±4.6)	0.049
Faecal pancreatic elastase (µg/g)	107.6 (±183.3)	153.2 (±211.2)	0.369
Faecal calprotectin (µg/g)	623.1 (±835.1)	96.9 (±87.4)	0.048

Data are shown as mean (±standard deviation). Elexacaftor–tezacaftor–ivacaftor (ETI).

**Table 5:** Quality of life questionnaires (CFQ-R) and Hospital Anxiety and Depression Scale.

	Pre ETI (*n* = 31)	Post ETI (*n* = 31)	*p*-Value
HADS_A_	4.9 (±3.5)	4.6 (±3.6)	0.640
HADS_D_	2.9 (±2.2)	2.5 (±3.2)	0.520
CFQ-R role	87.0 (±15.0)	89.0 (±16.8)	0.606
CFQ-R vitality	67.3 (±19.1)	76.3 (±19.5)	0.032
CFQ-R emotion	84.5 (±13.7)	84.3 (±15.5)	0.943
CFQ-R social	76.9 (±13.6)	82.0 (±15.7)	0.082
CFQ-R physical	65.8 (±20.7)	70.2 (±27.3)	0.344
CFQ-R body	68.9 (±20.8)	77. 8 (±18.4)	0.041
CFQ-R eatings	88.5 (±17.4)	94.2 (±11.2)	0.048
CFQ-R treatment	54.7 (±21.3)	67.1 (±20.2)	0.017
CFQ-R health	60.5 (±19.0)	75.1 (±20.1)	0.009
CFQ-R weights	66.7 (±37.3)	81.3 (±27.4)	0.046
CFQ-R respiratory	61.3 (±1.9)	85.3 (±1.4)	<0.001
CFQ-R digestion	76.5 (±17.4)	78.2 (±16.2)	0.621

Data are shown as mean (±standard deviation). Elexacaftor–tezacaftor–ivacaftor (ETI); CFQ-R: Cystic Fibrosis Questionnaire—Revised. Application; HADSA: Hospital Anxiety and Depression Scale, anxiety subscale; HADSD: Hospital Anxiety and Depression Scale, depression subscale.

## Data Availability

The original contributions presented in the study are included in the article, and further inquiries can be directed to the corresponding author due to privacy reasons.
